# Development of an Interview Guide Identifying the Rehabilitation Needs of Women from the Middle East Living with Chronic Pain

**DOI:** 10.3390/ijerph121012043

**Published:** 2015-09-25

**Authors:** Viktoria Zander, Henrik Eriksson, Kyllike Christensson, Maria Müllersdorf

**Affiliations:** 1 Department of Women’s and Children’s Health, Karolinska Institutet, Elevhemmet H2:00, Karolinska University Hospital Solna, Stockholm S-171 76, Sweden; E-Mails: viktoria.zander@dll.se (V.Z.); kyllike.christensson@ki.se (K.C.); 2 Centre for Clinical Research Sörmland, Uppsala University. Kungsgatan 41, Eskilstuna S-631 88, Sweden; 3 Department of Nursing and Care, the Swedish Red Cross University College, Teknikringen 1, Stockholm S-114 28, Sweden; E-Mail: henrik.eriksson@rkh.se; 4 School of Health, Care, and Social Welfare, Mälardalen University. P.O. Box 325, Eskilstuna S-631 05, Sweden

**Keywords:** immigrants, women’s health, primary healthcare

## Abstract

The purpose of this study was to develop an interview guide for use by primary healthcare professionals to support them in identifying the rehabilitation needs of forced resettled women from the Middle East living with chronic pain. Previous findings together with the existing literature were used as the basis for developing the interview guide in three steps: item generation, cognitive interviews, and a pilot study. The study resulted in a 16-item interview guide focusing on patients’ concerns and expectations, with consideration of pre-migration, migration, and post-migration factors that might affect their health. With the help of the guide, patients were also invited to identify difficulties in their daily activities and to take part in setting goals and planning their rehabilitation. The current interview guide provides professional guidance to caretakers, taking a person-centered participative point of departure when meeting and planning care, for and together, with representatives from dispersed ethnic populations in Sweden. It can be used together with the patient by all staff members working in primary healthcare, with the aim of contributing to continuity of care and multi-professional collaboration.

## 1. Introduction

As dispersed ethnic populations in Sweden grow, healthcare must consider specific needs that may arise from experiencing the trauma of war and forced resettlement, after being forced to leave one’s home and to settle in another part of the world. The largest proportion of people living in Sweden born outside of Europe is from the Middle East, of which most come from Iraq [1]. Researchers have reported that immigrants have worse self-reported health than the general population and experience difficulties in meetings with representatives from healthcare [2,3]. A study among Iraqis in Sweden has reported worse living conditions and health, together with a higher consumption of healthcare compared to the general population. In addition, almost half of the respondents stated that they did not seek healthcare despite needing it. Among these persons, trust in primary healthcare was low [2]. The complex situation of women from the Middle East diaspora living in Sweden and suffering from chronic pain makes it difficult to adapt healthcare to meet their needs. An interview guide could support healthcare professionals in identifying their needs in relation to the consequences of chronic pain in resettled women in Sweden.

Flight, asylum, and adjusting to life in a strange country are all components of an arduous process. Pre-migration experiences, the migration, and post-migration difficulties have both mental and physical consequences [2,3,4,5,6,7,8,9,10]. Many resettled people suffer from persistent or chronic musculoskeletal pain, which affects their bodies, their everyday lives, and their families and ultimately leads to a feeling of a loss of control [10,11]. 

The negative effects of forced resettlement have been described as being more pronounced for women than for men [12] because women are discriminated against both as women and as migrants. They have experienced violence and the consequences of war and, as a result, have unique physical, mental, economic, and social issues that affect their health [13]. Their experiences of forced resettlement together with intersectional structures such as gender, ethnicity, and age affect their opportunities in the host society and contribute to their vulnerability. In particular, there are reports of fatigue, homesickness, and somatic symptoms such as pain among these women [10,14].

Persons suffering from chronic pain have reported difficulties finding accessible, effective, and acceptable care, and they seek greater validation of their lived experiences of pain [10,15,16,17]. Their expectations of healthcare involve the need for diagnosis, the need to find effective treatments, and the need to keep the pain at a tolerable level. They also need helpful advice and information concerning pain management. 

With regard to the forced resettled, the experienced trauma may be challenging to healthcare professionals who are unfamiliar with the clinical consequences of refugee trauma [11]. There have been reports that even though healthcare professionals within Swedish primary healthcare identified the causes of pain among women from the Middle East to be trauma, loss, and grief, the measures they suggested were not aimed at these causes [18]. These results suggest difficulties in identifying and tailoring treatment to meet the needs of individuals suffering the consequences of cumulative trauma, in the shape of chronic pain. 

Refugee trauma and the consequences of torture are a highly topical subject in the Swedish media at present. It has been reported that although as many as about one-third of asylum seekers in Sweden have experienced torture, ignorance about physical and mental consequences is widespread within healthcare, social services, and schools [19,20]. 

There is a lack of evidence-based literature supporting effective treatments for resettled populations [11]. Previous research has argued for adapting assessments and treatments to the specific needs of resettled people [21] and that existing tools may be insufficient [7]. The complexities of meeting with women who have experienced cumulative trauma in the shape of chronic musculoskeletal pain suggest a need for increased support for healthcare professionals [18]. To the authors’ knowledge, there are no existing specific instruments that assess the rehabilitation needs of resettled women suffering from musculoskeletal pain that also consider the trauma the women may have experienced and that are easily administered in the clinical setting of primary healthcare. The aim of this study was to develop an interview guide for healthcare professionals within primary healthcare, to support the identification of the rehabilitation needs of women from the Middle East living with chronic pain. The aim of such a guide is to indicate the need for further assessment and to facilitate goal-setting.

The current study is part of a doctoral dissertation regarding rehabilitation of chronic pain in women from the Iraqi diaspora in Sweden. However, because most healthcare professionals seldom know the origin of their patients, the aim of this study was broadened to include women from the Middle East.

## 2. Materials and Methods

The interview guide was developed in three steps: item generation, cognitive interviews, and a pilot study. According to Streiner [22], instruments may be empirically based on a review of previous research or on new research. Items for the current interview guide were generated from the results of two previous studies [10,18] and a literature search. A literature search was conducted in PubMed, Cinahl and Web of Science using the following search terms: needs, needs assessment, healthcare needs, chronic pain, rehabilitation, immigrants and refugees. The review resulted in 52 articles. Of these, 20 were excluded due to the following exclusion criteria: studies focusing on medication substances, surgical treatment, or diagnostic specific (pancreatitis, cancer, asthma, *etc.*), in-patient treatment, labor migration, or asylum seekers. Thematic content analysis, as proposed by Burnard [23], was used to group the data into themes from which the items were drawn. The items were chosen to cover the dimensions of each theme.

After constructing the first draft of the guide, cognitive interviews [24] using think-aloud methods of the content were performed. Representatives from different professions in a health center in a medium-sized city in Sweden, with a high proportion of patients from the Iraqi diaspora, were asked to participate. Seven interviews were performed with two nurses, one physician, two physiotherapists, one occupational therapist, and one therapist. They all had experience meeting people from the Middle East in their everyday work. As a method, cognitive interviewing is used to identify possible response errors that may occur, for example, because of misunderstanding the questions [24]. The aim of the interviews was to understand how the questions were perceived and interpreted and to identify potential problems that might arise. This approach enabled the understanding of the items in the instrument from the user’s perspective rather than the researcher’s [24]. The interviews were conducted by the first author (Viktoria Zander) in a health center. Each participant was continuously encouraged throughout the interview to read the questions aloud and to say out loud every thought that came into their minds. The participants’ comments were recorded, transcribed, presented, and discussed in the research group and were the basis for revising the guide.

**Figure 1:**
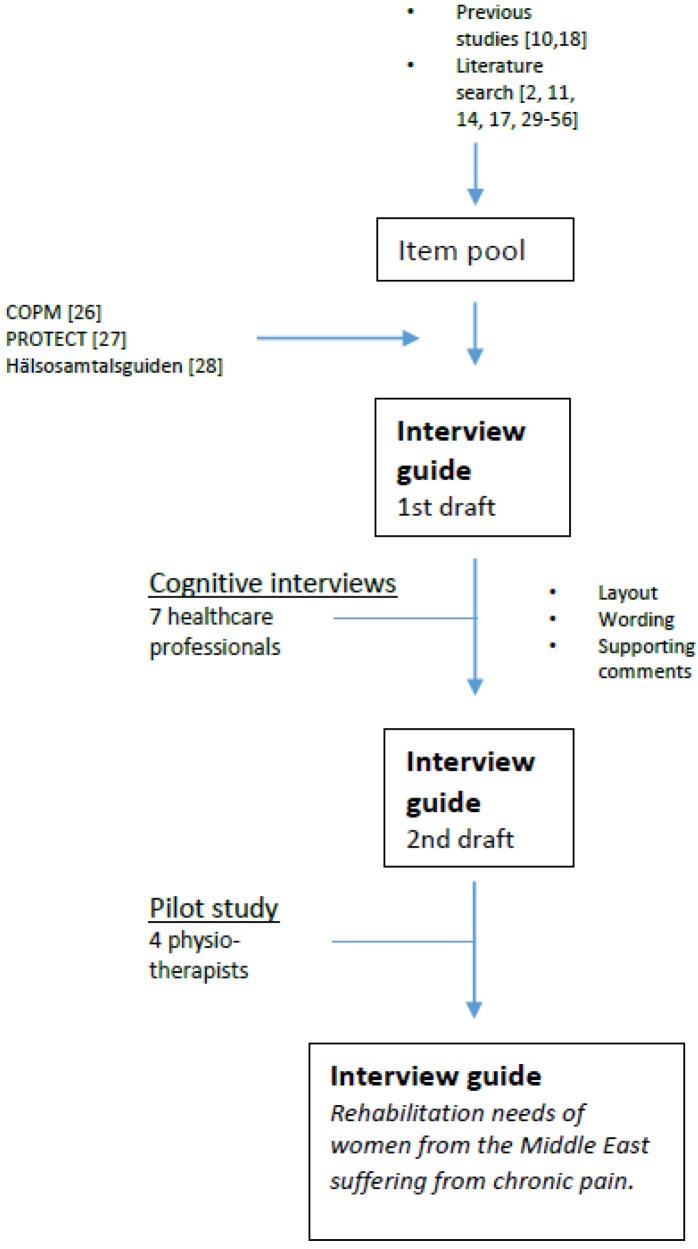
Development of the interview guide.

Representatives from primary healthcare who had participated in a previous study [18] were told about the interview guide and asked to participate in a pilot study of the instrument. Four physiotherapists consented to participate. No representatives of other professions showed any interest in participating. The physiotherapists worked in three different health centers in municipalities with a high proportion of people from the Iraqi diaspora. The purpose of the study was to evaluate the instructions, interpretation, and relevance of the items. They were informed about the instrument orally and in writing and were asked to use it for a five-week interval when meeting with women from the Middle East who were accessing primary care for chronic pain. At the end of the period, they were asked to reflect on and score the usefulness, item interpretation, and content of the instrument and its instructions [25] on a four-step Likert scale. The answers were sent to the first author. The participants’ reflections were presented to the research group and used to adapt the instrument. One of the participating physiotherapists gave no specification as to how many patients were tested with the interview guide. The others had tested it with between one and two patients each. The process is described in Figure 1.

The study was approved by the regional ethics committee in Stockholm, Sweden (2009/1819-31/2, 100111).

## 3. Results

### 3.1. Item Generation

Together with the results from two previous studies dealing with the consequences of chronic pain for Iraqi women [10,18], the 32 articles from the literature review formed an item pool that was divided into themes concerning (a) the consequences of pain; (b) the patient’s background; (c) the patient’s life situation; (d) pain management; and (e) the meeting with healthcare professionals. From these five themes, items were selected to form the first version of the interview guide. The items were also inspired by three existing instruments: the Canadian Occupational Performance Measure (COPM) [26], a method for assessing individual perceptions of problems in activities; the PROTECT questionnaire—questionnaire and observations for early identification of asylum seekers having suffered traumatic experiences [27], developed in a European Union project with the purpose of facilitating early recognition of persons having suffered traumatic experiences; and the “Health conversation guide” (Hälsosamtalsguiden) [28], developed by the Red Cross, Save the Children and the Church of Sweden to map the psychosocial needs of individuals and families who have just arrived in Sweden. The Canadian Occupational Performance Measure (COPM) [26] has shown acceptable test-retest reliability in different studies. It has also been proven valid in various areas of rehabilitation. The PROTECT tool [27] is a 10-item instrument developed to identify persons who have been exposed to psychological trauma. It is based on scientific knowledge of the psychological consequences of trauma and was developed by an interdisciplinary team of experts in asylum and the rehabilitation of torture survivors. The items have been chosen to take into account the most significant symptoms of psychological trauma. The third instrument, the “Health conversation guide” [28], was developed based on clinical evidence and in consultation with experts in refugee health. It was piloted, implemented, and evaluated for three years in southern Sweden. It was received positively by different organizations working with the integration of immigrants and refugees.

### 3.2. Cognitive Interviews

Seven interviews were performed resulting in adjustments regarding clarification of the wording, composition, and layout of the guide. No items were removed. The order of the items and the layout were changed to allow for space for written notes. Supportive comments for each item were added in the margins to act as a reminder about what was important to note in the patient’s answers and to clarify the purpose of the item. Responses to questions rating the patient’s perception of performance of an activity and satisfaction with this performance were changed from numeric scales to three-step Likert scales. This was done because the interviews revealed difficulties among the patients in evaluating a numeric scale from 0 to 10. A three-step Likert scale was perceived to be more suitable.

### 3.3. Pilot Study

The overall impression among the four physiotherapists in the pilot study was that the instructions and questions were comprehensive and relevant, but in general, the instrument was perceived as time-consuming and therefore difficult to implement in everyday work. During the pilot study, the participants were instructed to use the guide in their first meeting with the patient. Some of the participants thought it might be more useful to split the guide into two parts and use some of the questions in a later meeting. This was particularly related to questions about experiences of trauma because those could be perceived as difficult to discuss before being able to form a relationship and build trust with the patient.

### 3.4. Construction of the Interview Guide

The interview guide was constructed for use by all representatives within primary healthcare involved in pain rehabilitation. The questions are in Swedish but are intended to be used with an interpreter when needed. After revision, it contains 16 open-ended questions dealing with current problems/reasons for seeking healthcare (item 2), patient background including any previously experienced traumas (items 3 and 9), current life situation (items 7 and 8), thoughts about the future (items 10 and 11), medical history (items 4–6), and difficulties in everyday life (items 12–16). Based on the woman’s perception of the consequences of pain in her daily life and her strategies to manage these consequences, she is asked to prioritize three daily activities that are difficult to manage because of pain. Overall, this is the basis for setting goals and planning her rehabilitation. The focus is on the patient’s narrative, thereby inviting her to participate in the process. The interview guide items are presented in Table 1.

**Table 1:** The items selected for the interview guide regarding the rehabilitation needs of women from the Middle East suffering from chronic pain.

Number	Item	Comments	References
1.	Does the patient need an interpreter?		[10,11,29,30,31,32,33,34]
2.	What are the patient’s reasons for seeking healthcare?	The patient’s narrative. Listen for 3 minutes for beliefs, concerns, and expectations.	[10,35,36,37,38,39,40,41,42,43,44,45,46]
3.	The patient’s background?	Country of origin, family, previous occupation, reasons for migration, experienced trauma.	[10,11,17,18,28,39,47]
4.	What medical assessments has the patient gone through in Sweden or another country?	The patient’s narrative. Compare with beliefs, concerns, and expectations.	[10,17,18,29,30,33,37,45]
5.	What diagnoses has she received?		
6.	What treatments has she undergone?		
7.	What is the patient’s life situation in Sweden?	Living conditions, occupation, finances, social networks, satisfaction with her life.	[2,10,14,18,28,35,37,44,48,49,50,51]
8.	Is there anything or anyone in the patient’s surroundings that impacts her wellbeing?		[10,14,28,35,37,52,53]
9.	Does the patient experience: Sleeping difficulties? Nightmares? Persistent headaches? Easily getting angry? Thoughts about painful past events? Feelings of fear? Memory difficulties? Trouble concentrating?	Yes or no. Positive answers indicate risk for patient being traumatized.	[11,14,17,27,33,44,54,55,56]
10.	What plans does the patient have for her future?		[10,28]
11.	What does she need to do in order to fulfill her plans?		
12.	Experiences of difficulties in daily life?	Activities in everyday life, at work, leisure activities.	[10,17,26,39,56]
13.	How does the patient manage everyday life?	Active, passive, spiritual, and emotional strategies.	[10,17,18,35,39,53]
14.	Prioritized activities: 1. 2. 3.	Let the patient select three activities in her everyday life that she perceives as difficult to perform and that are important to her.	[26]
15.	Performance of prioritized activities?	Estimate performance: Not able to perform Can partially perform Is able to perform	
16.	How satisfied is the patient with her performance?	Estimate satisfaction with performance: Not satisfied at all Somewhat satisfied Fully satisfied	

## 4. Discussion

The result from this study is an interview guide that contains 16 questions that ask about the patient’s reasons for seeking healthcare, her background and previous experiences, the woman’s current life situation, thoughts about the future, how the woman perceives her medical history, and difficulties and strategies in everyday life. These questions are also the basis for setting goals. The current instrument was developed based on research about needs in relation to the consequences of chronic pain, in particular, in relation to forced resettlement among women from the Middle East. Although much of the evidence is consistent with evidence from individuals suffering from chronic pain in general, the women’s background of flight and resettlement contributes to the vulnerability of this specific target group. 

The basis of the interview guide is a person-centered approach, involving the patient in the decisions that are made, building a partnership, and documenting what has been decided [57]. 

Involving the patient in setting goals and planning the rehabilitation has been emphasized for chronic pain patients [58] and was confirmed by the literature search in regard to resettled populations. Based on that, the purpose of the current interview guide is to involve the woman in setting goals by focusing on her beliefs, concerns, and expectations and letting her identify and prioritize daily activities that are difficult for her to perform because of pain. 

The involvement of the patient stresses sufficient communication and, thus, the use of an interpreter when needed. The open-ended questions in the interview guide facilitate discussion based on the patient’s narrative. Inviting the person to speak, listening to her narrative, and finally summarizing what she has said are part of a person-centered and evidence-based method for interviewing [59] and a method to confirm sufficient communication. The informants in a previous study [10] reported that not having sufficient language abilities led to dependency on others in order to understand and to be understood. It contributed to their sense of not having control of their life situation. It also affected their meetings with healthcare providers. This was confirmed by other studies, showing that linguistic problems in several cases have led to misconceptions, and a lack of treatment, as well as mistreatment [60]. The items in the interview guide are in Swedish in order to be easily administered by Swedish-speaking healthcare professionals. As in all healthcare meetings, a professional interpreter is to be used when necessary. In these cases, the healthcare professional’s sensitivity to cultural differences is important, and they must always be aware of the risk of misunderstandings. 

With regard to the complex situation of the women, the guide should help map and document the patient’s specific needs related to pain. It takes a holistic approach, acknowledging physical, psychological, social, and spiritual components, which also implies the need for multi-professional collaboration together with the patient while still keeping the woman in control of the decisions that are made. Moreover, among chronic pain patients, there have been reports of the perception that healthcare professionals have not been able to meet the patients’ needs, as well as a lack of validation of their experiences of pain [15]. Because the encounter is conducted in the health professional’s arena, the patient is at a disadvantage. We argue that an approach similar to the approach suggested validates the patient’s experiences and identifies and adjusts the rehabilitation to his or her healthcare needs and expectations, taking into account intersectional power structures such as gender, age, and ethnicity.

Hence, the interview guide developed in the current study is a support for the healthcare professionals in identifying the needs of the woman, but also support for the woman by keeping her in power in the meeting.

Results from two previous studies, one qualitative [10] and one consensus [18], together with a literature review contributed to the content validity of the instrument. The face validity of the current interview guide was tested in cognitive interviews and in a pilot study in primary healthcare. Because this is an interview guide with mainly open questions, other dimensions of validity or reliability have not been tested. The pilot study revealed that although the questions were perceived as important and relevant, the guide was time-consuming and difficult to implement considering the time constraints in primary healthcare. However, the authors believe that in order to be able to plan rehabilitation for persons suffering from experienced trauma of forced resettlement and living with chronic pain, there is a need for an extensive assessment, even though it may be time-consuming. In such instances, the interview guide might function as support for a holistic approach, particularly as it is based on results from research on the experiences of both the women and healthcare professionals. 

In response to the time issue, the pilot study participants suggested dividing the interview guide into two meetings, meaning that some of the questions would be used in a later meeting. Specifically, separating questions about trauma was recommended because these would raise emotions and the re-experiencing of traumatic events among the patients. Leaving these questions to a later meeting, when mutual trust has been established, would make it easier for both the patient and healthcare professional. Using the interview guide as a mutual tool within the healthcare team would also mean that the patient would only have to tell her story at one single visit, which is favorable compared to experiences today in which patients are forced to retell, and relive, the story at every single visit to the health center [10].

The main limitation of this study was the small number of participants in the pilot study. The aim was to include representatives from different professions in primary healthcare, although in the end, only four physiotherapists consented to participate. During this study, we found that questions regarding rehabilitation were primarily referred to physiotherapists. Although the purpose was to develop an instrument suitable for different professions within primary healthcare, the experience was that it was difficult to involve professionals other than physiotherapists in the guide’s testing process. The purpose was also to contribute to multi-professional collaboration. The result from the pilot study raises the question of whether rehabilitation is considered to be solely the physiotherapist’s responsibility. Indeed, this is an onerous responsibility considering the complexity of chronic pain in forced resettled populations.

## 5. Conclusions and Future Implications

Based on previous research, the authors have identified a need to support and increase knowledge among healthcare professionals regarding involving patients and taking into consideration pre-migration, migration, and post-migration factors that might affect their health. Furthermore, there is a need for guidance and a person-centered participative point of departure when meeting and planning care, for and together, with representatives from dispersed ethnic populations in Sweden. Meeting these challenges was the reason for developing an interview guide that would support primary healthcare professionals in identifying the rehabilitation needs of resettled women from the Middle East suffering from chronic pain. The current guide is person-centered and may be used by all representatives from primary healthcare, together with an interpreter when needed. The guide invites the woman to verbalize trauma that too often is ignored by healthcare professionals, thus providing the prerequisites for a partnership in setting goals and planning rehabilitation. Its purpose is also to contribute to the continuity of care and multi-professional collaboration. In this manner, first and foremost, the interview guide is a person-centered tool to meet the specific challenges that healthcare professionals encounter as a result of the expanding ethnic populations in Sweden, challenges that, considering the ongoing conflicts in the world, will continue to exist and grow.

Whether the intention of the interview guide, *i.e.*, to support the identification and subsequently directly measure a patient’s individual needs, is fulfilled will be tested in future studies. The next step will be a test of responsiveness, the degree to which the interview guide serves its purpose as a support tool for healthcare professionals.
